# Fiber in Diet Is Associated with Improvement of Glycated Hemoglobin and Lipid Profile in Mexican Patients with Type 2 Diabetes

**DOI:** 10.1155/2016/2980406

**Published:** 2016-04-10

**Authors:** Lubia Velázquez-López, Abril Violeta Muñoz-Torres, Carmen García-Peña, Mardia López-Alarcón, Sergio Islas-Andrade, Jorge Escobedo-de la Peña

**Affiliations:** ^1^Unidad de Investigación en Epidemiología Clínica, Hospital “Carlos MacGregor Sánchez Navarro”, Instituto Mexicano del Seguro Social, Avenida Gabriel Mancera No. 222, Colonia del Valle, Delegación Bénito Juárez, 03100 Ciudad de México, Mexico; ^2^Departamento de Salud Pública, Facultad de Medicina, Universidad Nacional Autónoma de México, 6 Piso, Edificio B, Circuito Interior, Ciudad Universitaria, Avenida Universidad 3000, Ciudad de México, Mexico; ^3^Departamento de Investigación, Instituto Nacional de Geriatría, Periférico sur No. 2767, Colonia San Jerónimo Lídice, Delegación Magdalena Contreras, 10200 Ciudad de México, Mexico; ^4^Unidad de Investigación Médica en Nutrición, Hospital de Pediatría, Centro Medico Nacional “Siglo XXI”, Instituto Mexicano del Seguro Social, Avenida Cuauhtémoc 300, Colonia Doctores, Delegación Cuauhtemoc, 06720 Ciudad de México, Mexico; ^5^Unidad de Investigación Científica de Endocrinología, Diabetes y Metabolismo, y de Enfermedades Metabólicas, Centro Médico Nacional Siglo XXI, Instituto Mexicano del Seguro Social, Avenida Cuauhtémoc 300, Colonia Doctores, Delegación Cuauhtemoc, 06720 Ciudad de México, Mexico

## Abstract

*Objective.* To assess the association of dietary fiber on current everyday diet and other dietary components with glycated hemoglobin levels (HbA1c), glucose, lipids profile, and body weight body weight, in patients with type 2 diabetes.* Methods.* A cross-sectional survey of 395 patients with type 2 diabetes was performed. HbA1c, fasting glucose, triglycerides, and lipids profile were measured. Weight, waist circumference, blood pressure, and body composition were measured. Everyday diet with a semiquantitative food frequency questionnaire was evaluated. ANOVA, Kruskal-Wallis, chi-square tests and multivariate logistic regression were used in statistical analysis.* Results.* Higher fiber intake was associated with a low HbA1c, high HDL-c levels, low weight, and waist circumference. The highest tertile of calories consumption was associated with a higher fasting glucose level and weight. The highest tertile of carbohydrate consumption was associated with a lower weight. The lowest tertile of total fat and saturated fat was associated with the highest tertile of HDL-c levels, and lower saturated fat intake was associated with lower weight (*p* < 0.05).* Conclusions.* A higher content of fiber in the diet reduces HbA1c and triglycerides, while improving HDL-c levels. Increasing fiber consumption while lowering calorie consumption seems to be an appropriate strategy to reduce body weight and promote blood glucose control.

## 1. Introduction

Increased fiber in the diet is associated with a reduction of glycated hemoglobin (HbA1c), improved lipid profile, and loss of body weight in type 2 diabetes patients. It has been proposed that appropriate consumption of fruit in the diet may be an adequate strategy to reduce HbA1c, given the fiber content, and prevent complications from diabetes [1, 2]. Several clinical trials have documented a positive effect of dietary fiber, [3] and in cross-sectional studies this effect has also been observed in different Asian populations [1, 4]. In fact, dietary fiber can reduce the incidence of stroke in patients with type 2 diabetes [5].

There is evidence of the benefit of reduced calorie diets, though the combination of this restriction with a diet higher in protein to encourage weight loss and control glucose and serum lipid levels continues to generate controversy [6]. It has also been suggested that reducing the proportion of carbohydrates in the diet at the cost of increasing protein consumption improves HbA1c and blood pressure values as well as weight loss and this, combined with physical exercise, proffers greater protection [7]. The recommendation to include fats in the diet continues to stir up controversy regarding the effect of this on the lipids profile and body weight; strategies include reducing to 30% or less the calorie intake. Conversely, higher consumption has been suggested though with a higher content of monounsaturated and polyunsaturated fats [8].

Type 2 diabetes is one of the chronic diseases with the highest economic and social impact, as well as in the repercussions on the quality of life. In recent decades it has reached epidemic proportions; lifestyle is a very important factor in the prevention and control of the disease [9]. There is evidence of the favorable effect of adequate glycemic control to prevent micro- and macrovascular complications [10]. In the treatment regime, diet adaptation is a basic element to achieve and maintain control goals [11]. Even while diet plays a substantial role, only a minority of patients receive nutriment therapy, and an even smaller number undergo the needed changes in diet as part of their disease management [12].

Fiber consumption used to be high in the Mexican population; however, it has been reduced due to changes in eating habits [13] which undoubtedly affects habitual diet in subjects with diabetes. While there is limited information on the description of current everyday diet in patients with type 2 diabetes and its effect on major cardiovascular risk factors, the role of macronutrients on metabolic control indicators continues to foster controversy [14]. Given the foregoing, the objective of this study was to assess the association of habitual dietary fiber and other dietary components on HbA1c, glucose, and lipid profile levels, as well as body weight, in type 2 diabetes patients in Mexico.

## 2. Methods

This report corresponds to the baseline evaluation of type 2 diabetes patients that were included in a multicenter, randomized clinical trial called Efficacy of Nutritional Therapy and Education through a Multimedia System for the Metabolic Control of Type 2 Diabetes Patients (ClinicalTrials.gov identifier: NCT02441023). Patients came from four different primary care clinics (family medical units belonging to the Mexican Social Security Institute) in Mexico City, with a previous diagnosis of diabetes, with age younger than 70 years, and with no advanced microvascular complication. An ethics and research committee approved the protocol. Patients were invited to participate at their local clinics and incorporated into the study once they had signed the letter of informed consent.

The patients included in the study underwent an extensive medical history questionnaire as well as a physical examination to evaluate the presence of severe complications. Hypertension was diagnosed when the patient stated being on antihypertensive medication or for those with values ≥140/90 mmHg at the time of joining the study. Blood pressure was taken twice with a mercury sphygmomanometer at an interval of 4 minutes between measurement and after the patient had been seated for more than 5 minutes. The mean of the two values was used. Physical activity was considered when the patient had at least 150 min/week of moderate-intensity aerobic physical activity, for at least 3 days/week with no more than 2 consecutive days without exercise [15].

### 2.1. Variables Measured

Twelve-hour-fasting blood plasma, glucose concentration, creatinine, triglycerides, total cholesterol, and fractions HDL-c values were measured; the LDL-c was calculated through the Friedewald equation LDL-c = [total cholesterol − (triglycerides/5) − HDL-c, for triglyceride concentration < 400 mg/dL] [16]. HbA1c was determined through the high-resolution liquid chromatography method. Excretion of urinary albumin was analyzed in the first morning urine sample through photometry/nephelometry to determine the albumin-creatinine ratio.

Standardized nutritionists who employed the Habitch method and the specifications recommended by Lohman recorded anthropometry [17, 18]. Waist circumference was measured after determining the midpoint between the last rib and the upper edge of the iliac crest on the right side; hip circumference was measured at the widest point of the trochanters. Both of these measurements were taken on three occasions, and the mean of the second and third measurement was used for the purpose of analysis. The percentage of fat was obtained through the bioimpedance measurement of the lower segment, using a TANITA*™* body composition analyzer, model TBF-215.

Diet was assessed with a food frequency questionnaire, previously validated and currently used in the Mexican National Health and Nutrition Surveys [19, 20]. The frequency with which 116 food items and 8 types of beverages were consumed in the last year was measured. Frequency was described as never in the last year or frequency of consumption over a one-month, one-week, or one-day period in the last year. Included on the list were dairy, eggs, meat and cold cuts, fruit, vegetables, legumes and cereals, sweets, drinks, and fats to prepare or dress foodstuffs.

In order to calculate the consumption of nutrients we employed the software* System to calculate nutritional vectors*, developed by the National Public Health Institute of Mexico (INSP in Spanish). Dietary components analyzed were consumption of calories, proteins, carbohydrates, and lipids, as well as fiber content, cholesterol, and sodium in the diet. The consumption of nutriments was analyzed, with an adjustment of the total calories using the residual method in order to remove the effect of energy consumption of nutriments [21].

### 2.2. Statistical Analysis

For statistical analysis, frequency and proportion measurements were calculated, as were the mean and standard deviation to characterize the population in sociodemographic variables, disease history, and proportion of metabolic control.

A one-factor analysis of variance was performed to identify the association between the main dietary components with metabolic control variables; the Kruskal-Wallis test was used for glucose and triglyceride variables. The chi-square test was used to identify the association between a balanced diet and the various components of metabolic control.

A logistic regression multivariate analysis was performed to identify the risk of HbA1c >7% if the dietary components were inadequate. The model was adjusted with the variables that could influence metabolic controls, such as the number of years elapsed since the time of diagnosis, the type of treatment given, and diabetes education. Data were considered statistically significant with a *p* < 0.05. Statistical analysis was done using the SPSS package, version 19.

## 3. Results

The average age of the study population was 54.6 ± 8.0 years; oral hypoglycemic drugs were the most-used diabetes treatment (76%); a low proportion followed a diet and physical activity (16% and 13%, resp.). Only 21% were prescribed lowering lipid medication. Of the study group, 55% had hypertension, 26% had diabetic neuropathy, 12% had microalbuminuria, and 5% had macroalbuminuria (Table 1).

Table 2 shows the metabolic control data and the dietary characteristics of the patients. Only 34% had a desirable HbA1c; total cholesterol was within control range for over 50% of the population, whereas triglycerides, LDL-c, and HDL-c were found to be within normal range in only third of the subjects. Body mass index (BMI) was adequate in only 12%; half of the population was obese and 37% of the population was overweight; over 70% of patients had an abnormal waist circumference. Diastolic blood pressure rather than systolic showed uncontrolled values (43%). Forty-five percent consumed a diet with appropriate calorie content for their current weight, and proteins are the dietary macronutrient showing the highest degree of appropriate consumption, while 48% of the study population was found to have an appropriate fiber consumption.

Table 3 shows the association between the main dietary components and the metabolic control indicators. There is a noticeable positive association between high calorie consumption and glucose levels, while lower consumption of total and saturated fat was associated with higher levels of HDL-c. A higher intake of fiber in the diet was significantly linked to lower levels of HbA1c and triglycerides and HDL-c improvement.

The association between the components of the diet and the anthropometric indicators is presented in Table 4. A greater consumption of calories in the diet was associated with greater weight, whereas a higher consumption of total carbohydrates produced a reverse association with weight gain. Lower consumption of saturated fat is associated with less weight, whereas a higher fiber intake is associated with lower levels of weight and waist circumference.

In the multivariate analysis (Table 5), a lower intake of dietary fiber (first quartile) was independently associated with poor glycemic control, together with longer duration of the disease and lack of education in diabetes. In a similar model, the lowest tertile of fiber consumption increased the risk of inadequately low HDL-c levels, as did the higher the body weight, but the effects of duration of diabetes and education in diabetes were lost (Table 6).

## 4. Discussion

In patients with type 2 diabetes, adherence to an appropriate meal plan and physical activity are important components in controlling the disease. Of the population assessed, only 16% of patients followed a diet as part of their treatment and only 13% exercised. As for metabolic control, only 3 out of 10 patients had HbA1c <7%, a proportion similar to what is reported in the National Health Survey of Mexico 2012, in which only a small group adheres to diet and exercise regimes as part of treatment, and only 24% have an adequate glycemic control [22].

Regarding the characteristics of the diet, only 2 out of 10 patients have adequate consumption of whole simple carbohydrates, only 16% consume adequate proportions of fat, and only 8% have a proper balance in their daily diet, even though 4 out of 10 patients are consuming a diet conducive to attaining healthful body weight (1,200 to 1,600 calories), calories suggested if the individual is sedentary, with overweight or obesity, and more than 50 years old. Other authors have proven the association between obesity and a diet high in calories, saturated fat, sodium, and a lower content of fiber in type 2 diabetes patients [23]. The results shown are consistent with the association between higher calorie consumption and greater glycemic and weight instability. A very small proportion of patients exhibit a proper balance in diet; thus it is necessary to encourage them to adhere to a more healthful alimentary regime.

An increased consumption of carbohydrates was linked significantly to lower weight. No differences were found regarding the consumption of carbohydrates and glucose or HbA1c levels, similar to what has been observed by other authors when comparing the effect of prescribing diets with a greater or lesser carbohydrate content, on HbA1c levels and body weight [24]. However, a review of clinical trials where patients were followed up for more than six months confirms the benefit of prescribing reduced carbohydrate diets, of low glycemic index, Mediterranean diets, and diets with higher protein content to improve cardiovascular risk markers in patients with type 2 diabetes [25]. The jury is still out for a real consensus regarding the distribution of macronutriments in the diet of the diabetic patient. In fact, consensus reached on the treatment of the disease suggests that diet should be tailored and adjusted to the comorbidity of each patient [11]. The effect that the amount of carbohydrates in the diet has on body weight and glycemic levels merits further evaluation and monitoring. It is well known that one of the main health problems linked to diabetes is obesity, reported to be as high as 50% in this population, and obesity elevates the risk of macrovascular disease. The habitual diet of patients is one of the most important factors to reduce obesity. It is imperative to design educational strategies geared toward improving adherence to a healthful diet.

In this study, consumption of proteins was not associated with improved glycemic control. Other authors have reported the benefits of higher protein content in the diet for an improvement of HbA1c and body weight, as well as some cardiovascular risk indicators [26]. Different theories regarding the effect of a high protein diet have been described, including the benefit of producing greater satiety and reducing consumption of carbohydrates—which provide less energy—leading to weight loss. On the other hand, the higher thermal effect of proteins versus carbohydrates fosters greater weight loss and improved metabolic control in overweight or obese patients [27].

As for fat in the diet, high consumption of saturated fat is linked to higher risk of cardiovascular disease, obesity, and dyslipidemia when compared to a higher consumption of polyunsaturated fat. In our results, increased consumption of saturated fat was linked to lower levels of HDL-c and higher body weight. These results are consistent with the broadly described benefits of the Mediterranean diet, in which the quality of fat in the diet is what leads to a reduction of cardiovascular risk indicators. Results encourage higher consumption of poly- and monounsaturated fat and a reduction of at least 10% of saturated fat in the total calorie intake [28].

The association between dietary fiber consumption and improved HbA1c, HDL-c, and weight levels is consistent with what has been reported by other authors regarding the effect of fiber in reducing postprandial glucose, increased satiety, better glycemic control, improvement of cardiovascular risk factors, and reduced risk of macrovascular complications [29, 30]. Even though, in the studied population, 48% had an adequate dietetic fiber consumption (14 g/1000 calories), it is still important to promote a varied diet, with a high consumption of high soluble and insoluble fiber foods, mainly derived from vegetables, whole grains, dried fruits, and fruits with a low glycemic index.

An important component for controlling the diabetes is the education; only three out of every ten patients assessed have been in an education program. Our results show the protective effect that this strategy provides to aid the attainment of improved HbA1c levels, added to what has been reported regarding the prevention of micro- and macrovascular complications [31]. Despite the foregoing, education continues to be an underused approach; we must insist on its use as a measure to promote a healthful lifestyle and a better care of the disease. Comprehensive treatment should be aimed at obtaining adequate metabolic control and at encouraging the detection and timely medical and nutritional treatment of diabetes [32].

## 5. Conclusions

A higher content of fiber in the diet had an impact on reducing HbA1c and triglycerides, while improving HDL-c levels. Lower calorie and saturated fat consumption may still be an appropriate strategy to reduce body weight, promote blood glucose control, and improve HDL-c levels. Since metabolic control is deficient in Mexicans, we must insist on a higher fiber content in the diet, given the beneficial effects noted in bringing down HbA1c levels and weight, while improving lipid profile.

## Figures and Tables

**Table 1:** Sociodemographic characteristics and comorbidity in type 2 diabetes patients.

*n* = 395	*n* (%)
Age	54.6 ± 8.0^*∗*^
Sex	
Female	270 (68)
Male	125 (32)
Diagnosis of diabetes (years)	6 (3–11)^*∗∗*^
Diabetes treatment	
No pharmacological treatment	16 (4)
Oral hypoglycemic agents	300 (76)
Oral hypoglycemic agents and insulin	43 (11)
Insulin	36 (9)
Lipid lowering medication	84 (21)
Follow a diet plan	65 (16)
Physical activity	51 (13)
Education in diabetes	129 (33)
Hypertension	219 (55)
Smoking	89 (22)
Alcohol consumption	132 (33)
Neuropathy	
Peripheral	77 (20)
Autonomic	22 (6)
Kidney disease
Glomerular filtration rate <60 mL/min/1.73 m^2^	18 (5)
Microalbuminuria 30–299 mg/g	49 (12)
Macroalbuminuria >300 mg/g	18 (5)

Data are presented as frequencies and percentage. ^*∗*^Age is presented as mean ± standard deviation. ^*∗∗*^Years of diagnosis of diabetes are presented as median and interquartile range.

**Table 2:** Metabolic control and diet characteristics of the studied population (*n* = 395).

	Mean ± SD or median (Interquartile range)	Reference value	Adequate control *n* (%)
Biochemical variables			
HbA1c (%)	8.48 ± 2.2	<7%	136 (34)
Fasting glucose (mg/dL)	146 (117–201)	70–130 mg/dL	149 (38)
Total cholesterol (mg/dL)	195.90 ± 41.1	<200 mg/dL	229 (58)
LDL-c (mg/dL)	112.46 ± 32.0	<100 mg/dL	128 (32)
HDL-c (mg/dL)	41.68 ± 11.2	>50 F and >40 M mg/dL	70 (25.9)/41 (33)
Triglycerides (mg/dL)	180 (134–251)	<150 mg/dL	134 (34)
Creatinine (mg/dL)	0.81 ± 0.2	0.7–1.3 mg/dL	387 (98)
Anthropometric variables and blood pressure (BP)			
Weight (kg)	75.42 ± 14.8	—	—
Body mass index (kg/m^2^)	30.52 ± 5.3	18.5–24.9 kgm^2^	47 (12)
Waist circumference (cm)	100.48 ± 12.4	<80 (F) and <90 (M) cm	12 (4)/24 (19)
Body fat (%)	41.90 ± 11.7	20–30 (F) and 12–20 (M) cm	5 (4)/12 (4)
Systolic BP (mmHg)	124.50 ± 15.9	≤130 mmHg	324 (83)
Diastolic BP (mmHg)	83.25 ± 11.1	≤80 mmHg	224 (57)
Diet variables			
Total energy kcal/day^1^	1792.25 ± 683.2	20–30 kcal/kg/day	179 (45)
Carbohydrates (g/day)/%	225.65 ± 93.6/**50.3 ± 7.8**	50–55%	97 (25)
Soluble CHO g/day	59.00 (37.87–70.81)/**13.1 ± 4.5**	<10%	97 (25)
Proteins g/day/%	67.59 ± 23.3/**15.5 ± 3.0**	10–15%	188 (48)
Fats g/day/%	70.02 ± 28.8/**36.0 ± 7.0**	25–30%	64 (16)
Dietary fiber g/1000 kcal	13.83 ± 0.4	14 g/1000 Kcal	188 (48)
Saturated fats g/day/%	20.24 ± 9.4/10.1** ± 2.2**	<7%	28 (7)
Fructose g/day	20 (14–27)	(20 g)/day	195 (49)
Cholesterol mg/day	195 (129–252)	<200 mg	212 (54)
Sodium mg/day	1492.57 ± 594.0	<2000 mg	269 (82)
Balanced diet^2^	—	—	30 (8)

^1^Caloric recommendations for adults older than 51 years and sedentary behavior were considered, as well as a caloric reduction for subjects with diabetes, obesity, or overweight; in normal weight, 30 kcal/kg/day was considered in reference value, in overweight, 25 kcal/kg/day, and, in obesity, 20 kcal/kg/day. ^2^Balanced diet was considered when the proportion of carbohydrates was between 50 and 55%, proteins 10–15%, and fats < 30% from total kcal value. F: female. M: male.

**Table 3:** Association of diet components and metabolic control variables (*n* = 395).

Tertiles	HbA1c %	Glucose mg/dL	Triglycerides mg/dL	HDL-c mg/dL	LDL-c mg/dL
Calories (kcal)					
812–1440	8.40 ± 2.1	140.0 (116.2, 189.7)	181.0 (131.0, 252.0)	41.8 ± 10.6	113.9 ± 31.2
1441–1922	8.35 ± 2.3	141.0 (112.2, 193.5)	171.5 (135.2, 235.0)	41.1 ± 9.7	111.7 ± 31.5
1922–3420	8.70 ± 2.3	157.0 (123.0, 224.0)	182.0 (135.0, 267.0)	42.2 ± 13.1	111.6 ± 33.2
*p* value	*0.400*	***0.0130***	*0.603*	*0.719*	*0.811*
Carbohydrates (g/day)					
116.45–211.36	8.6 ± 2.1	141.0 (116.5, 215.5)	186.0 (141.5, 251.5)	40.6 ± 10.0	108.0 ± 32.0
211.37–238.80	8.4 ± 2.3	145.0 (113.0, 200.0)	174.0 (130.5, 243.0)	41.4 ± 13.4	116.1 ± 33.2
238.81–298.50	8.4 ± 2.3	148.5 (119.0, 189.7)	168.5 (128.2, 247.0)	43.1 ± 10.1	112.5 ± 29.2
*p* value	*0.767*	*0.882*	*0.254*	*0.202*	*0.117*
Proteins (g/day)					
40.72–63.06	8.5 ± 2.3	152.0 (121.5, 198.0)	168.0 (131.0, 255.5)	42.1 ± 9.7	117.9 ± 30.11
63.07–70.36	8.7 ± 2.4	151.0 (114.0, 211.5)	189.0 (138.7, 242.5)	42.0 ± 12.9	110.3 ± 31.8
70.37–98.24	8.2 ± 2.0	139.0 (113.0, 199.0)	179.0 (136.0, 252.0)	41.2 ± 11.05	110.21 ± 33.3
*p* value	*0.166*	*0.196*	*0.418*	*0.776*	*0.080*
Fats (g/day)					
12.92–64.50	8.5 ± 2.3	148.0 (119.0, 195.0)	174.0 (130.0, 257.0)	43.9 ± 13.3	112.3 ± 29.0
64.51–74.77	8.5 ± 2.3	152.0 (119.0, 203.0)	179.0 (137.0, 266.5)	40.1 ± 10.1	114.4 ± 34.7
74.78–149.02	8.4 ± 2.1	140.0 (111.0, 216.0)	182.0 (141.0, 235.0)	41.6 ± 9.7	110.6 ± 31.1
*p* value	*0.859*	*0.627*	*0.779*	***0.017***	*0.633*
Saturated fats (g/day)					
1.18–18.20	8.3 ± 2.1	146.0 (115.0, 194.0)	165.0 (125.0, 236.0)	43.9 ± 9.9	116.8 ± 28.2
18.21–21.89	8.7 ± 2.4	145.0 (119.0, 205.0)	184.0 (141.0, 270.0)	40.0 ± 9.8	112.9 ± 34.6
21.90–54.28	8.5 ± 2.2	149.0 (116.0, 209.0)	183.0 (138.0, 238.0)	41.1 ± 13.4	107.6 ± 31.7
*p* value	*0.260*	*0.968*	*0.096*	***0.015***	*0.065*
Dietary fiber (g/day)					
4.63–21.65	8.6 ± 2.3	151.5 (119.5, 208.5)	183.0 (142.8, 251.8)	42.0 ± 12.8	114.5 ± 31.5
21.66–26.68	8.8 ± 2.3	152.0 (116.0, 221.0)	186.0 (133.0, 284.0)	39.5 ± 9.0	111.0 ± 33.7
26.69–78.38	8.0 ± 2.1	136.5 (114.0, 186.8)	163.0 (123.5, 221.0)	43.5 ± 11.2	111.8 ± 30.5
*p* value	**0.011**	0.203	**0.007**	**0.016**	0.622

Data are presented as mean ± standard deviation or median (interquartile range), according to each tertile of diet components. Comparison was performed with ANOVA (means) or Kruskal-Wallis (medians).

**Table 4:** Association of diet components with anthropometric and body composition variables.

Tertiles	Weight (kg)	Waist circumference (cm)	Body mass index (kg/m^2^)	Body fat (%)
Calories (kcal)				
812–1440	72.6 ± 13.6	99.4 ± 12.2	29.8 ± 5.0	42.4 ± 11.1
1441–1922	76.4 ± 16.0	100.9 ± 12.6	31.0 ± 5.5	41.9 ± 11.3
1922–3420	77.3 ± 14.6	101.2 ± 12.6	30.8 ± 5.3	41.4 ± 12.9
*p* value	***0.022***	*0.458*	*0.145*	*0.782*
Carbohydrates (g/day)				
116.45–211.36	78.1 ± 16.0	101.4 ± 12.5	30.8 ± 5.3	41.2 ± 11.3
211.37–238.80	74.2 ± 14.5	100.6 ± 12.4	30.4 ± 5.3	42.7 ± 12.3
238.81–298.50	73.8 ± 13.6	99.4 ± 12.4	30.2 ± 5.3	41.7 ± 11.7
*p* value	***0.035***	*0.416*	*0.654*	*0.572*
Proteins (g/day)				
40.72–63.06	75.9 ± 14.1	101.5 ± 12.2	30.9 ± 5.4	43.2 ± 11.9
63.07–70.36	74.5 ± 15.1	99.8 ± 12.4	30.2 ± 4.9	41.9 ± 11.1
70.37–98.24	75.8 ± 15.4	100.2 ± 12.6	30.4 ± 5.6	40.5 ± 12.2
*p* value	*0.705*	*0.528*	*0.538*	*0.172*
Fats (g/day)				
12.92–64.50	75.0 ± 14.5	101.0 ± 12.4	30.5 ± 5.4	40.9 ± 12.1
64.51–74.77	74.3 ± 14.1	99.7 ± 12.3	30.3 ± 5.2	42.6 ± 11.7
74.78–149.02	76.9 ± 15.9	100.7 ± 12.5	30.7 ± 5.3	42.1 ± 11.5
*p* value	*0.347*	*0.698*	*0.853*	*0.462*
Saturated fats (g/day)				
1.18–18.20	72.6 ± 13.5	99.2 ± 12.8	29.8 ± 5.2	40.9 ± 12.2
18.21–21.89	75.9 ± 15.4	100.4 ± 11.8	30.7 ± 5.3	42.7 ± 11.0
21.90–54.28	77.7 ± 15.3	101.8 ± 12.6	31.0 ± 5.3	42.0 ± 12.0
*p* value	***0.020***	*0.223*	*0.183*	*0.450*
Dietary fiber (g/day)				
4.63–21.65	78.1 ± 14.2	102.5 ± 12.2	31.0 ± 5.2	42.2 ± 12.5
21.66–26.68	74.1 ± 14.9	99.3 ± 11.5	30.1 ± 5.0	41.9 ± 10.9
26.69–78.38	73.9 ± 15.2	99.5 ± 13.3	30.4 ± 5.6	41.5 ± 11.9
*p* value	**0.037**	**0.063**	0.407	0.888

Data are presented as mean ± standard deviation, according to each tertile of diet components. Comparison was performed with ANOVA.

**Table 5:** Odds ratios (OR) and 95% confidence intervals (CI_95%_) derived from a multivariate logistic regression model to identify the risk of an inadequate glycemic control (HbA1c > 7%).

	OR	CI_95%_	*p* value
Total energy (kcal)			
812–1440	1		
1441–1922	0.81	0.46–1.41	0.459
1922–3420	1.35	0.75–2.41	0.309
Dietary fiber (g/day)			
26.69–78.38	1		
21.66–26.68	2.16	1.22–3.83	0.008
4.63–21.65	1.91	1.08–3.35	0.024
Years since diagnosis			
<5 years (reference)	1		
5–10 years	2.26	1.28–3.99	0.005
>10 years	2.01	1.01–3.78	0.029
Pharmacologic treatment			
Oral hypoglycemic drugs (reference)	1		
Oral hypoglycemic drugs and insulin	6.97	2.32–20.86	0.001
Insulin	5.28	1.70–16.35	0.004
No drugs	0.79	0.27–2.36	0.683
Education in diabetes			
Yes (reference)	1		
No	2.19	1.35–3.42	0.002
Sex			
Male (reference)	1		
Female	0.84	0.51–1.38	0.496
BMI			
Normal weight	1		
Overweight	1.05	0.49–2.25	0.890
Obesity	1.02	0.48–2.16	0.950

Inadequate glycemic control (HbA1c > 7%), odds ratios (OR), and 95% confidence intervals (CI_95%_). Calories and dietary fiber are presented in tertiles. BMI: body mass index. Individuals were considered of normal weight with BMI < 24.9, overweight with 25–29.9, and obesity > 30 kg/m^2^.

**Table 6:** Odds ratios (OR) and 95% confidence intervals (CI_95%_) derived from a multivariate logistic regression model to identify the risk of an inadequate HDL-c.

	OR	CI_95%_	*p* value
Total energy (kcal)			
812–1440	1		
1441–1922	1.029	0.56–1.83	0.977
1922–3420	0.87	0.47–1.58	0.640
Dietary fiber (g/day)			
26.69–78.38	1		
21.66–26.68	1.05	0.57–1.94	0.873
4.63–21.65	1.79	0.98–3.27	0.059
Years since diagnosis			
<5 years (reference)	1		
5–10 years	1.46	0.80–2.62	0.211
>10 years	1.13	0.60–2.12	0.701
Pharmacologic treatment			
Oral hypoglycemic drugs (reference)	1		
Oral hypoglycemic drugs and insulin	1.01	0.45–2.24	0.930
Insulin	0.65	0.29–1.46	0.300
No drugs	1.83	0.48–6.90	0.369
Education in diabetes			
Yes (reference)	1		
No	1.45	0.89–2.37	0.133
Sex			
Male (reference)	1		
Female	1.55	0.92–2.61	0.103
Fats (g/day)	1.018	1.00–1.03	0.046
12.92–64.50	1		
64.51–74.77	1.87	1.02–3.39	0.040
74.78–149.02	1.19	0.65–2.17	0.573
Saturated fats (g/day)			
1.18–18.20	1		
18.21–21.89	1.32	0.72–2.43	0.366
21.90–54.28	1.20	0.61–2.35	0.590
Physical activity			
Yes	1		
No	1.20	0.61–2.35	0.590
Peso Kg	1.017	1.00–1.03	0.042

Inadequate HDL-c (<40 mg/dL in male and <50 mg/dL in female), odds ratios (OR) and 95% confidence intervals (CI_95%_). Calories, dietary fiber, fats, and saturated fats are presented in tertiles.
